# SPiCE: a web-based tool for sequence-based protein classification and exploration

**DOI:** 10.1186/1471-2105-15-93

**Published:** 2014-03-31

**Authors:** Bastiaan A van den Berg, Marcel JT Reinders, Johannes A Roubos, Dick de Ridder

**Affiliations:** 1 Delft Bioinformatics Lab, Department of Intelligent Systems, Faculty Electrical Engineering, Mathematics and Computer Science, Delft University of Technology, Mekelweg 4, 2628CD, Delft, The Netherlands; 2, DSM Biotechnology Center, Delft, The Netherlands; 3, Netherlands Bioinformatics Centre, Nijmegen, The Netherlands; 4, Kluyver Centre for Genomics of Industrial Fermentation, Delft, The Netherlands

**Keywords:** Sequence-based, Data visualization and exploration, Protein feature extraction, Protein classification

## Abstract

**Background:**

Amino acid sequences and features extracted from such sequences have been used to predict many protein properties, such as subcellular localization or solubility, using classifier algorithms. Although software tools are available for both feature extraction and classifier construction, their application is not straightforward, requiring users to install various packages and to convert data into different formats. This lack of easily accessible software hampers quick, explorative use of sequence-based classification techniques by biologists.

**Results:**

We have developed the web-based software tool SPiCE for exploring sequence-based features of proteins in predefined classes. It offers data upload/download, sequence-based feature calculation, data visualization and protein classifier construction and testing in a single integrated, interactive environment. To illustrate its use, two example datasets are included showing the identification of differences in amino acid composition between proteins yielding low and high production levels in fungi and low and high expression levels in yeast, respectively.

**Conclusions:**

SPiCE is an easy-to-use online tool for extracting and exploring sequence-based features of sets of proteins, allowing non-experts to apply advanced classification techniques. The tool is available at http://helix.ewi.tudelft.nl/spice.

## Background

The sequence of a protein contains valuable information about its characteristics. Various sequence-based prediction methods exploit this to classify proteins according to specific properties, such as localization [1], function [2], or solubility [3]. This has resulted in relevant and frequently used bioinformatics tools [4] that are offered by a growing number of easily accessible websites and webservices [5-7].

**Figure 1:**
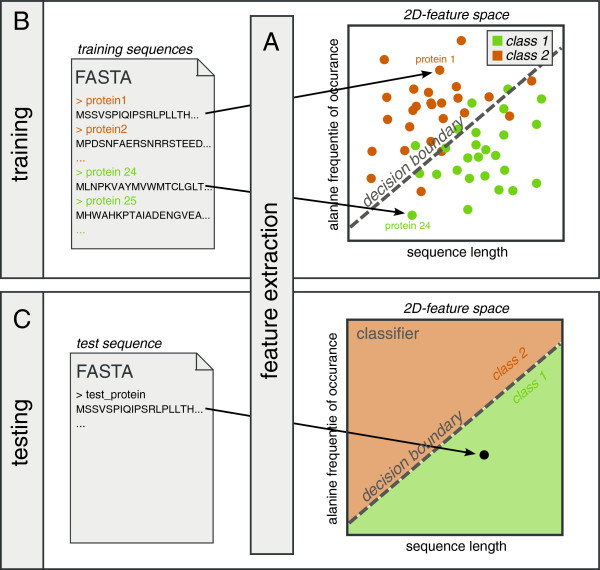
**Protein classification.A)** Feature extraction maps protein sequences to feature space. In this case, calculation of the sequence length (*x*-axis) and the relative frequency of occurrence of alanines (*y*-axis) map each protein sequence to a point in two-dimensional feature space. **B)** Classifier training using proteins with known class labels: class 1 (orange) and class 2 (green). After mapping to feature space, a classifier is trained to obtain a decision boundary (dashed line) that optimally separates the classes. **C)** Predicting class labels of new proteins using the trained classifier. After mapping to feature space, the point in feature space determines what label is assigned to the protein. Label class 1 will be assigned to the example protein, because of its location on the class 1 side of the decision boundary.

Sequence-based protein classifiers assign class labels to proteins based on a set of features, real numbers that capture some sequence property. This process entails three distinct steps. First, *feature extraction* is required to map protein sequences to points in a feature space (Figure 1A). Next, a classifier is constructed to optimally separate protein classes in this feature space (*training*, Figure 1B), using a set of proteins with known class labels. Finally, the trained classifier can be applied to predict class labels for new proteins (*testing*, Figure 1C). Additionally, features and feature distributions can be visualized to explore differences between protein classes by eye.

Software tools are available for each of these three steps. Feature extraction is available as software package [8] and through web services [9-12] and an extensive range of classification software has been developed [13,14], some of which include feature visualization [15]. However, combined application requires installing different software packages and programming efforts to convert data according to the requirements of each tool. For the construction of specialized high-performance classifiers, the overhead of deploying such a pipeline may be acceptable or even required, because this usually involves extensive exploration of many combinations of (customized) features, types of classifiers, and parameter settings. However, it precludes easy access to these methods for non-expert users.

We set out to offer basic protein classification functionality in a single environment to allow for quick and easy exploration of user-defined protein classes, without the need for any programming, data conversion or software installation. To this end we introduce SPiCE, a web-based tool for Sequence-based Protein Classification and Exploration. SPiCE makes powerful data exploration techniques accessible to non-experts; additionally, expert bioinformaticians can exploit the back-end software to perform customized and/or computationally expensive tasks on a local computer.

## Implementation

Before describing the SPiCE functionality, some classification concepts and the offered sequence-based features will be introduced in the following two sections.

### Classification

Classifiers are algorithms that assign discrete class labels to objects. These objects are typically represented as vectors of features, real numbers that reflect a property thought to be potentially different for proteins in the different classes. Protein sequences should therefore first be mapped onto such feature vectors, a process called *feature extraction* (Figure 1A). This should ideally result in a small number of discriminative features. In SPiCE, feature vectors are always normalized to zero mean and unit standard deviation.

Given a *training set* of proteins with known labels, a classifier can then be trained, i.e. its parameters can be tuned to yield optimal performance (Figure 1B). For problems with two classes *A* and *B*, performance is often estimated based on a receiver-operator characteristic (ROC) curve. Such a curve represents all possible trade-offs between classifications of proteins in class *A* as being in class *B* and vice versa. If class *A* corresponds to “positive” and class *B* to “negative”, the ROC curve is traditionally drawn as false positive rate vs. true positive rate and the area under the ROC curve (AUC) is used as a measure of classifier performance, with 1 indicating perfect classification and 0.5 random classification. Once trained, the trained classifier can be used to predict the class label for any new protein, a process called *testing* (Figure 1C).

To avoid overtraining, i.e. setting the parameters such that the training set is classified well but test samples will be classified poorly, a stratified cross-validation scheme is used. This entails splitting the training set in *k* parts reflecting the original class distributions (where the “fold” *k* is a parameter) and iteratively training classifiers on *k*−1 parts and estimating its performance on the remaining part. The average performance is then an estimate of the performance to be expected on new, unseen data.

A large number of classification algorithms are available, differing in complexity and often applicable to specific problems. SPiCE implements the most well-known classifier types (see Table 1). In case the classifier has parameters, they are optimized in an inner *k*-fold cross-validation loop [16] using the parameter ranges in Table 1 as search grid, optimizing for the AUC.

**Table 1:** Offered classifiers with corresponding parameter ranges

**Classifier**	**Parameter optimization grid**
SVM (linear kernel)	*C*=10^−3^,10^−2^,…,10^3^
SVM (RBF kernel)	*C*=10^−1^,10^0^,10^1^
	*α*=10^−1^,10^0^,10^1^
*k*-neighbors (unif.^1^)	*k*=1,2,…,5,10,20,…,50,100
*k*-neighbors (dist.^2^)	*k*=1,2,…,5,10,20,…,50,100
Nearest centroid	*r*=1,2,…,10
LDA^3^ classifier	-
QDA^4^ classifier	-
Gaussian Naive Bayes	-
Decision tree	*Default scikit-learn parameters*
Random forest	*Default scikit-learn parameters*

^1^uniform resp. ^2^distance-based neighbor weights, ^3^linear discriminant resp. ^4^quadratic discriminant analysis.

For a more in depth discussion of classification and feature extraction, the reader is referred to relevant reviews [17,18] or textbooks [19,20]. Below, an overview of the specific features SPiCE extracts from protein sequences is given.

### Sequence-based features

Table 2 lists the feature categories that can be calculated; these categories are briefly discussed below. More details can be found on the SPiCE documentation page (http://helix.ewi.tudelft.nl/spice/doc).

**Table 2:** Sequence-based feature categories

**Feature category**	**Parameters**	**Number of features**
***Composition features***		
AA composition ^∗^	Number of segments	20× number of segments
Dipeptide composition	Number of segments	400× number of segments
Terminal end amino acid count	N- or C-terminal end, length	20
SS composition ^∗^	Number of segments	3× number of segments
Per SS class AA composition ^∗^	-	3×20
SA composition ^∗^	Number of segments	2× number of segments
Per SA class AA composition ^∗^	-	2×20
Codon composition	-	64
Codon usage	-	64
Protein length	-	1
***Property profile-based features***		
Signal average	AA scale(s), window, edge	1 per AA scale
Signal peaks area	AA scale(s), window, edge, threshold	2 per AA scale
Autocorrelation	Type, AA scale(s), distance	1 per AA scale
Pseudo AA composition (type 1) ^∗^	AA scale(s), *λ*	20+*λ*
Pseudo AA composition (type 2) ^∗^	AA scale(s), *λ*	20+*λ*
***Amino acid distance-based features***		
Property CTD ^∗^	Property	21
Quasi-sequence-order	AA distance matrix, *λ*	20+*λ*

^*^AA: amino acid, SS: secondary structure, SA: solvent accessibility, CTD: composition, transition, distribution.

#### Composition features

These features calculate letter counts divided by sequence length for a number of sequence types: amino acid, codon, secondary structure, and solvent accessibility. The ‘number of segments’ parameter subdivides sequences into equal length parts and returns the composition of each segment separately. For the amino acid sequence, there is also the option to calculate the dipeptide composition, i.e. amino acid pair counts divided by sequence length −1, and the amino acid counts for a given length of the N- or C-terminal end of the protein sequence. For the codon sequence, the codon usage can be calculated.

#### Property profile-based features

Amino acid scales map each amino acid to a value that captures a physicochemical or biochemical property, such as hydropathicity or size. These scales are used to obtain a property profile for a protein sequence by mapping all of its residues to the corresponding values. The profiles are in turn used for calculating property profile-based features. The AAIndex data base [21] contains a large collection of scales that can be selected for feature calculation. Because the data base contains many correlated scales, a set of 19 uncorrelated scales derived from the entire AAIndex database [22] can also be selected. Amino acid scales are normalized (zero mean, unit standard deviation) before using them for feature calculation. 

•*Signal average* features capture, based on the selected amino acid scale used for generating a property profile, the average property over the entire protein sequence by calculating the average profile value.

•*Signal peaks area* features use the property profiles to capture occurrences of property peaks by calculating the sum of all areas under a protein profile above and below a given threshold. A window and edge parameter define the width and edge weights of a triangular filter with which the profile is convoluted to smooth it before calculating the features [23].

•*Autocorrelation* features employ the property profiles to calculate property correlations between two residues at a given distance over the entire protein sequence. As in profeat, three different types are implemented: normalized Moreau-Broto [24], Moran [25], and Geary [26].

•*Pseudo-amino acid composition* features calculate the amino composition with additional features that include sequence-order information up to a given distance *λ*. Sequence-order information is incorporated by calculating residue correlation factors between two residues at a given distance over the entire protein sequence, for distances 1,2,…,*λ*. The correlation factors are based on one or multiple user-defined amino acid scales as offered by the PseAAC web server [10]. Both the parallel-correlation type (type 1), as introduced in [27] for predicting protein cellular attributes, and the series-correlation type (type 2), as introduced in [28] for predicting enzyme subfamilies, are offered by SPiCE.

#### Amino acid distance-based features

These feature categories use amino acid distances for feature calculation, either by using a amino acid distance matrix or by using predefined amino acid clusters. 

•*Property composition, transition, distribution (CTD)* features were previously used to predict protein folding classes [29]. Our implementation is based on profeat[12]. The twenty amino acids are subdivided into three groups; A, B, and C, based on a given property. Protein sequences are then mapped to the reduced three-letter alphabet (ABC), which are used to calculate *i*) the property composition, letter counts divided by sequence length, *ii*) property transitions, the number of AB and BA transitions divided by the sequence length - 1 (likewise for AC and BC), and *iii*) the property distribution, relative protein sequence positions of the first occurrence, the 1^st^, 2^nd^, and 3^rd^ quantile, and the last occurrence of each property letter. The used properties – hydrophobicity, normalized Van der Waals volume, polarity, polarizibility, charge, secondary structures and solvent accessibility – and corresponding amino acid subdivisions are the same as in profeat.

•*Quasi-sequence-order descriptors* have been used to predict protein subcellular localization [30]. They are comparable to the pseudo amino acid composition, but the Schneider-Wrede amino acid distance matrix [31] is used for calculating correlation factors instead of amino acid scales.

### Functionality

**Figure 2:**
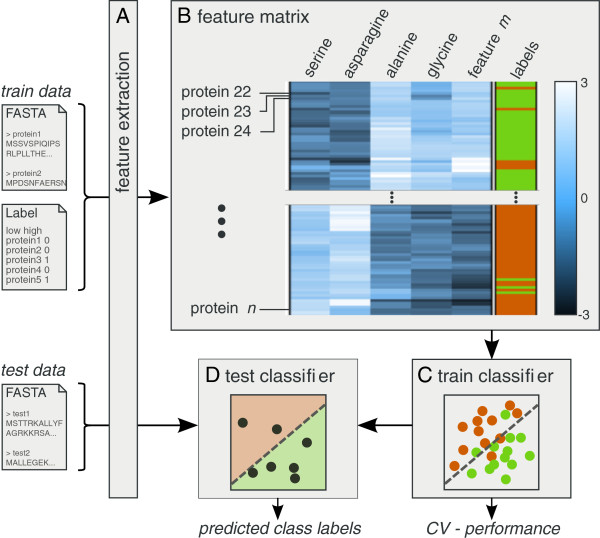
**Overview of the four main functionalities.A)** Sequence-based feature extraction, mapping each protein in a FASTA file to a list of feature values (a row in the feature matrix). The uploaded protein labels will be used for classifier construction. **B)** Visual inspection of the calculated feature data, in this example showing (part of) the feature matrix in the form of a clustered heat map with in each row the feature values of one protein and the corresponding protein labels in the rightmost column. **C)** Classifier construction using the calculated feature matrix and the provided labels (train data). A *k*-fold cross-validation protocol is used to assess classification performance. **D)** The trained classifier can be used to predict class labels for a set of new proteins (test data).

SPiCE has four main functionalities, as illustrated in Figure 2. First, users can upload a FASTA file with protein sequences for which a range of sequence based features can be calculated (Figure 2A). The resulting feature matrix (Figure 2B) can then be visually explored using histograms, scatter plots, and heat maps. Classifiers can be trained for a set of user-defined class labels (Figure 2C) and the resulting classifier can finally be used to predict class labels of new protein sequences (Figure 2D).

**Figure 3:**
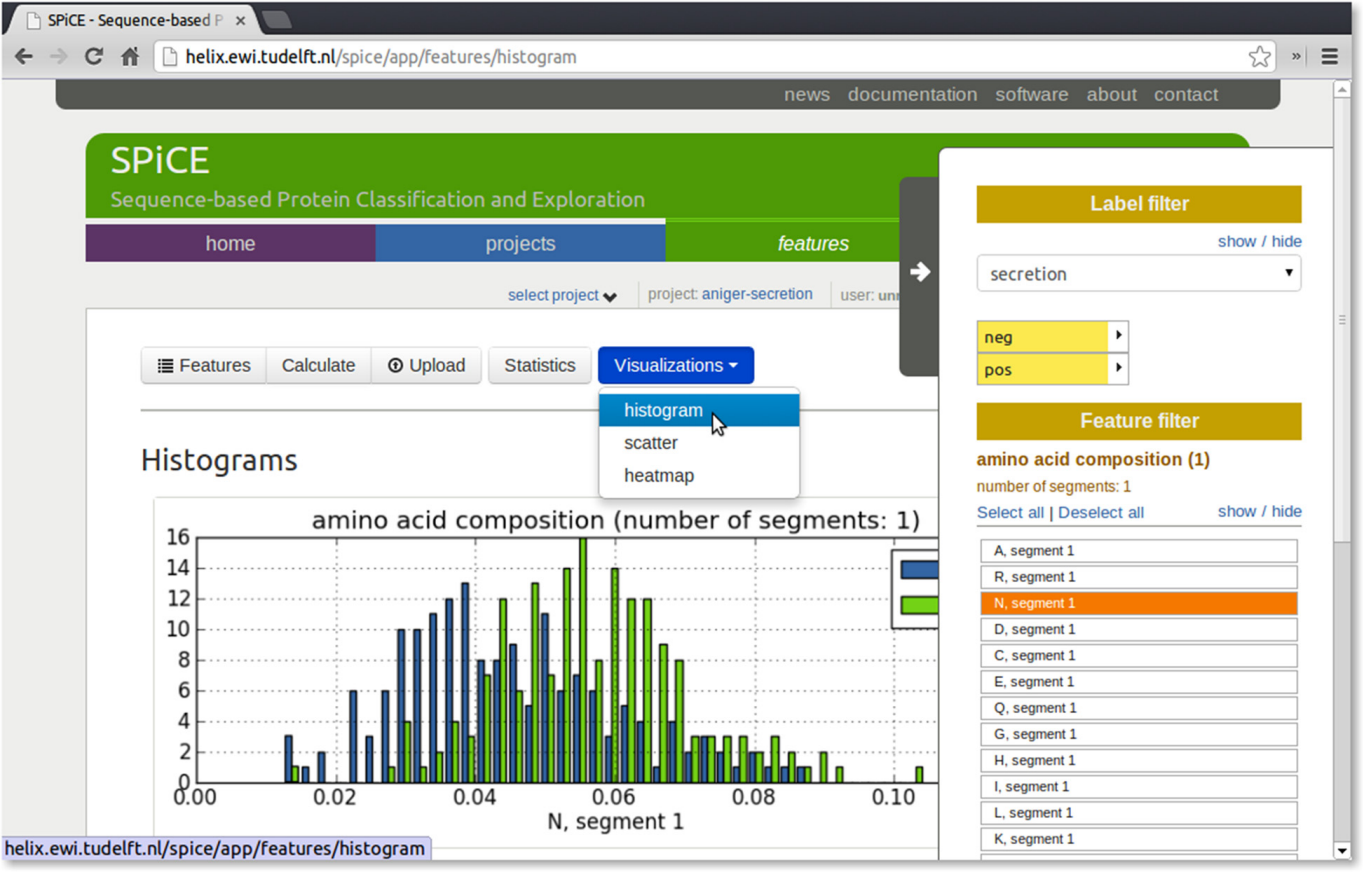
SPiCE screenshot.

To access these functions, the SPiCE web-based user interface offers four areas: *home*, *projects*, *features*, and *classification*, accessible through the main tabs. The web application can be freely explored without registration. A user account bar – situated directly underneath the main tabs (Figure 3) – enables users to login to their account or to create a new account, providing them with a secure personal work space in which their projects will be stored.

*Home* contains general information and news items. Additional documentation and tutorials can be accessed through the *documentation* link in the header menu at the top of the page (Figure 3).

*Projects* are initiated by uploading a FASTA file with either protein (amino acid) or ORF (nucleotide) sequences. After initiation, one or more labeling files can be uploaded in which each protein is assigned a label, for example its subcellular localization. Users can also upload (predicted) secondary structure and solvent accessibility sequences, which enables the calculation of additional features.

*Features* can be calculated for all proteins in the project. A list of available sequence-based features is given in Table 2. Additionally, users can upload their own calculated features. The resulting feature matrix can be explored using different visualizations. Feature-value distributions and class separation can be explored using histograms (e.g. like in Figure 3) and scatter plots. Another way of exploring predictive features is to visually inspect the feature matrix using a hierarchically clustered heat map (Figure 2B), in which the protein labels are added as an extra column (not used for clustering).

*Classification* offers the ability to train classifiers using the proteins in the current project. Users can select: *i)* the type of classifier to use, *ii)* the classes to train for, *iii)* the features to use for training, and *iv)* the number of cross-validation loops *k*. A (double) *k*-fold cross-validation protocol is used to assess classifier performance and to optimize classifier parameters if required. After training, a table with performance measures is reported, together with a receiver operating characteristic (ROC) curve in case of two-class classification. The final classifier is trained on the entire train set using the optimized parameter settings. Trained classifiers can be applied to predict class labels of new proteins by selecting any of the user’s projects, in which case class labels will be predicted for each protein in that project.

### Software framework

The website is developed in Python 2.7.3 (http://www.python.org), using the minimalist python web framework CherryPy 3.2.0 (http://www.cherrypy.org). The back-end uses the Python package *spice* for feature calculation and classification. Within this package, the *featext* module manages feature extraction using a *dataset* module to manage protein sequence data and a *featmat* module to manage the labeled feature matrix. The *classification* module offers a set of classification tasks, which basically is a layer on top of the machine learning software scikit-learn 0.14.1 [14]. Feature extraction and classification tasks are put in a job queue which is handled by a separate compute server.

## Results and discussion

To validate the system, we reproduced results of previous work in which a data set was employed to construct classifiers predicting successful high-level production of extracellular proteins in *Aspergillus niger*[32]. The used data set consists of 345 secretory proteins that were over-expressed in *A. niger* and tested for detectable extracellular concentrations by putting the obtained extracellular medium on a gel after growing the culture in shake flask. A label ‘high’ was assigned to proteins for which a band on the gel was observed and a label ‘low’ to the others, resulting in 167 high-level and 178 low-level proteins. This labeled protein set can be loaded as an example project in SPiCE.

**Figure 4:**
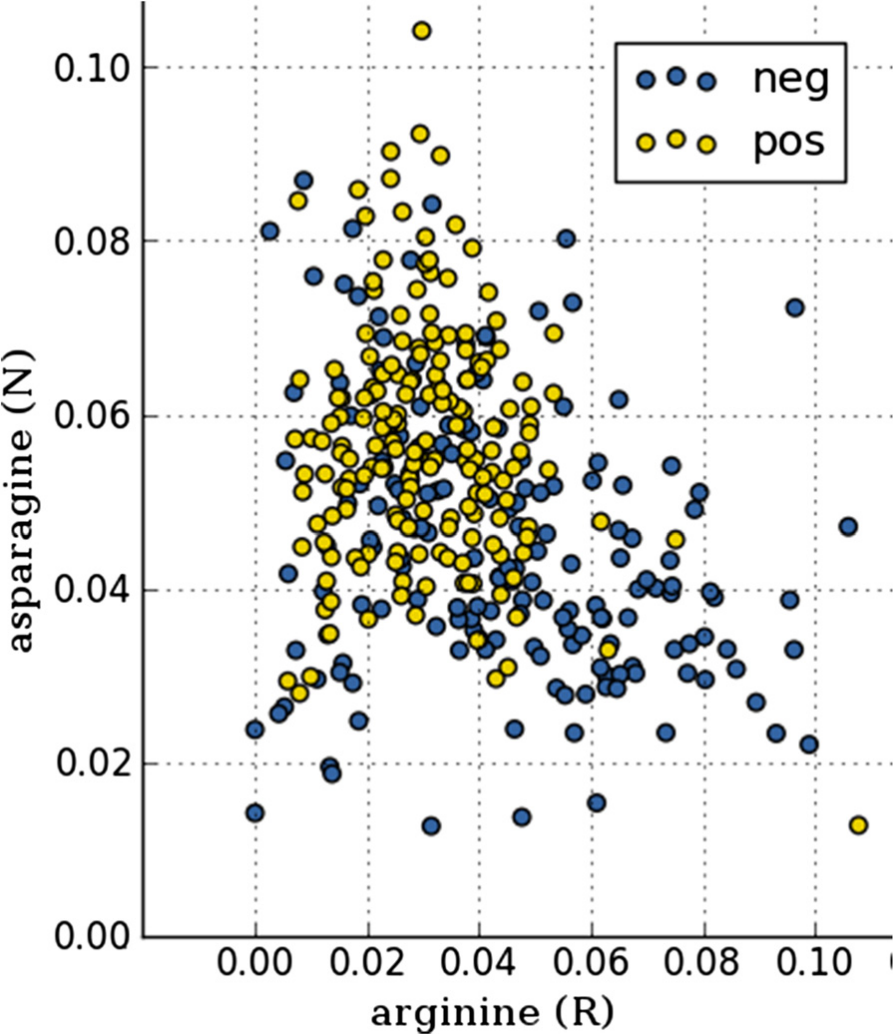
**Scatter plot showing class separation for the** ***A. niger* secretion project using the amino acid composition features with the lowest (negative) and highest** ***t*-value, arginine and asparagine respectively.**

**Figure 5:**
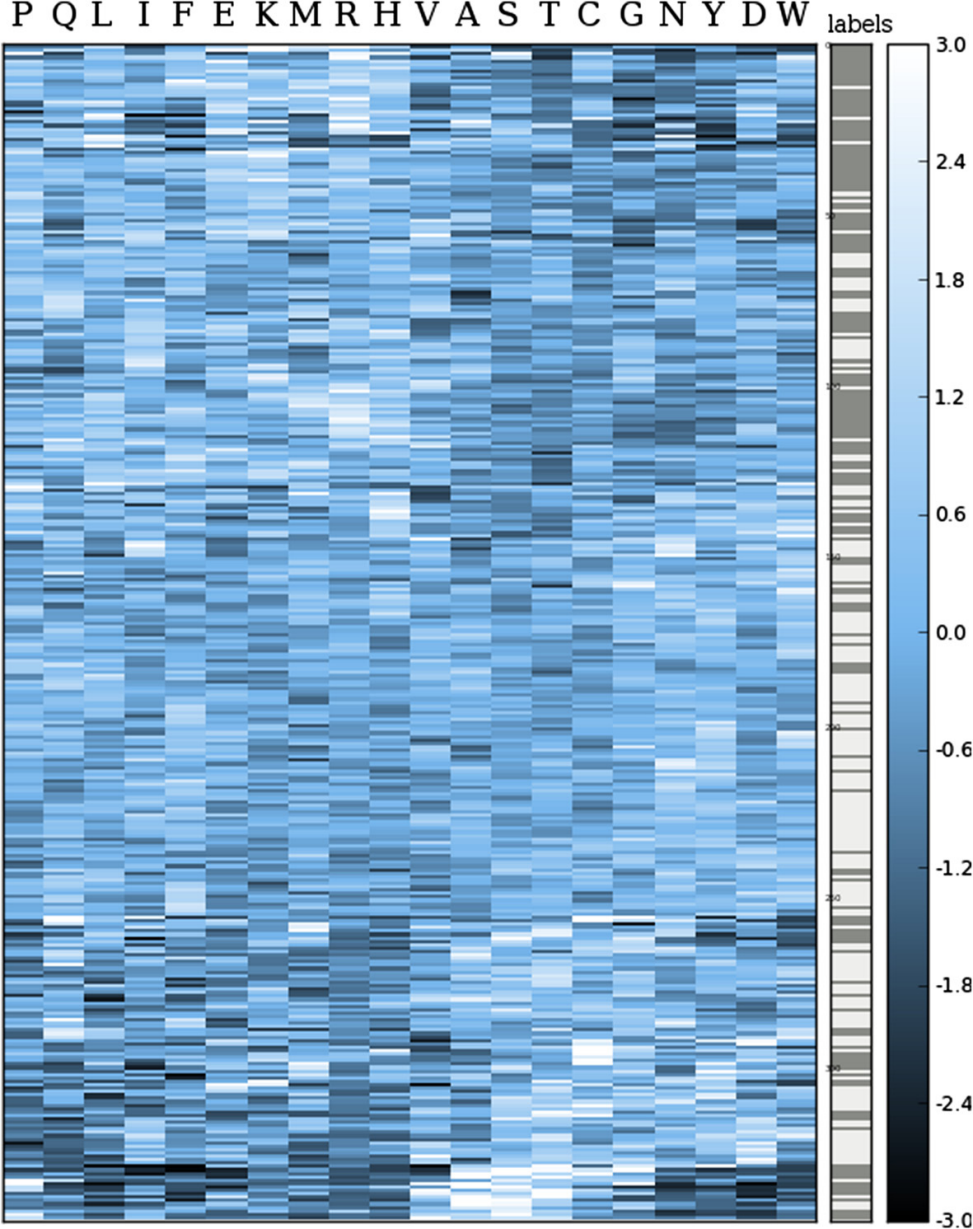
**Hierarchically clustered feature matrix of the*A. niger* secretion project with the amino acid composition features as columns and the proteins as rows.** The corresponding class labels, gray for ‘low’ and white for ‘high’, are shown in the column on the right.

The amino acid composition was calculated and used for the construction of a linear support vector machine (10-fold double-loop cross-validation), providing results that are in agreement with the results described earlier [32]. Similar to the observations in that work, the *t*-statistics reveal strong predictive capacity for the tyrosine, asparagine, arginine, and lysine features (Additional file 1: Figure S1), which can also be observed in the histograms (Additional file 1: Figure S2). The scatter plot in Figure 4 shows the obtained class separation by using the two features with the lowest (negative) and highest *t*-value respectively. For the hierarchically clustered feature matrix in Figure 5, clustering of proteins (rows) with the same label indicate that these features are useful for classification. Classifier construction resulted in a cross-validation performance of 0.837 area under the ROC curve (Additional file 1: Figure S4), again similar to results obtained in [32].

Additionally, we used a yeast protein expression data set to illustrate the ease with which one can explore differences between user-defined protein classes. For this data set, yeast proteins were split into low-level and high-level expressed based on data found in [33], in which *Saccharomyces cerevisiae* open reading frames were tagged with a high-affinity epitopes and expressed from their natural chromosomal location after which protein abundances were measured during log-phase growth by immunodetection of the tag. As a pre-processing step, to avoid a bias for sets with highly similar proteins, BLASTCLUST[34] was used to reduce sequence redundancy. After that the list of proteins was ordered by expression level. The top and bottom 1000 proteins were labeled ‘high’ and ‘low’ respectively. This data is also available as an example project.

**Figure 6:**
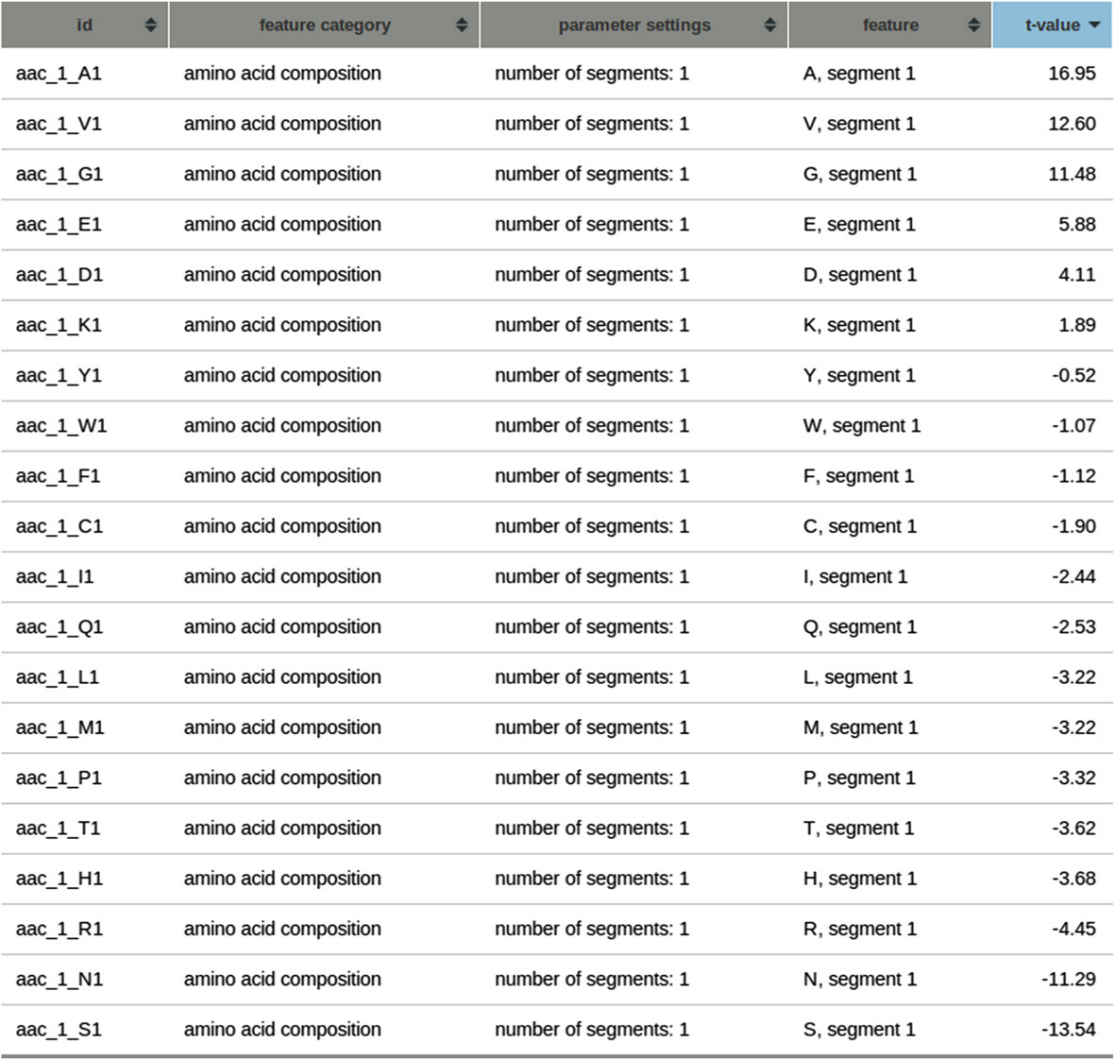
**Table with*t*-statistics of the yeast expression-level project.** The table shows the *t*-statistics for the amino acid composition features and is ordered by *t*-value. High absolute *t*-values indicate a difference in class means of the two (assumed normal distributed) class distributions.

**Figure 7:**
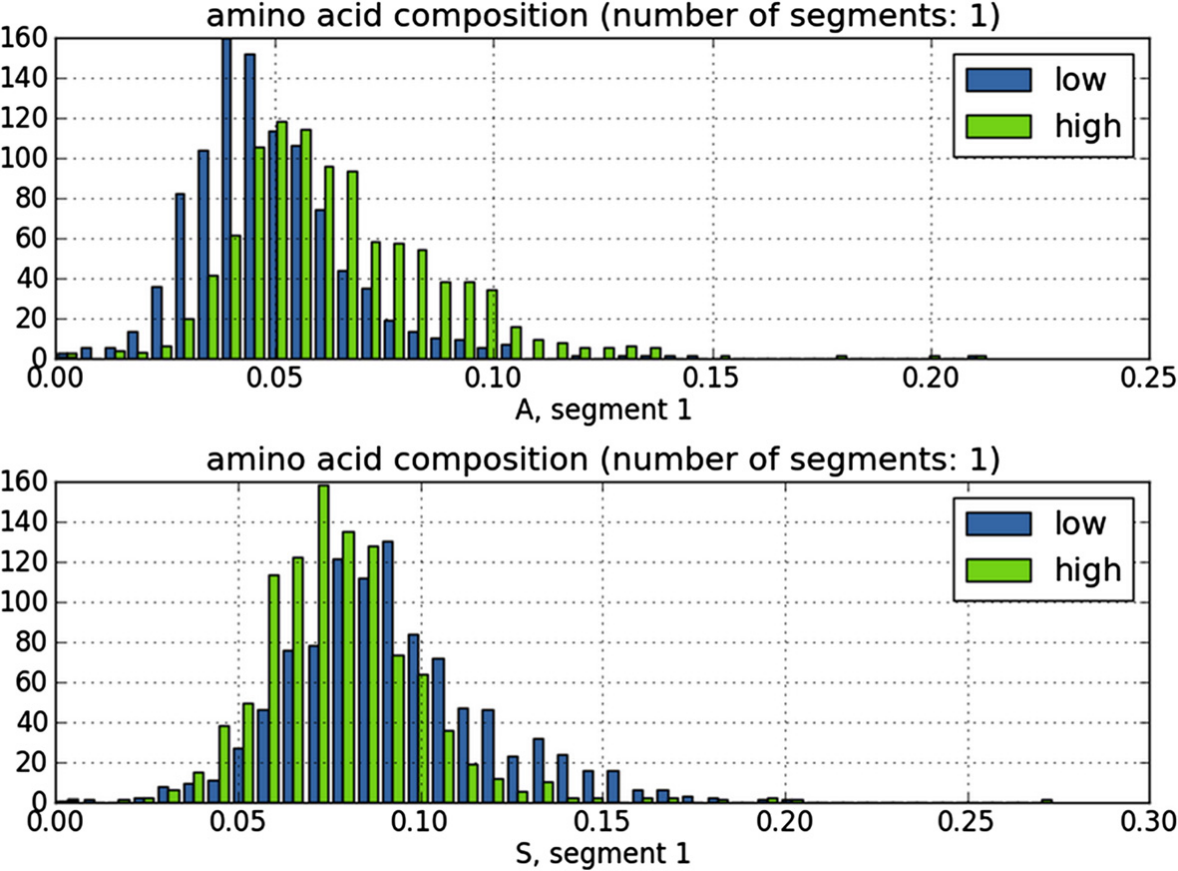
**Histograms of the yeast protein expression-level project.** Histograms are shown for the two amino acid composition features with largest positive and negative *t*-values (Figure 6), alanine and serine respectively, showing different means of the class distributions.

**Figure 8:**
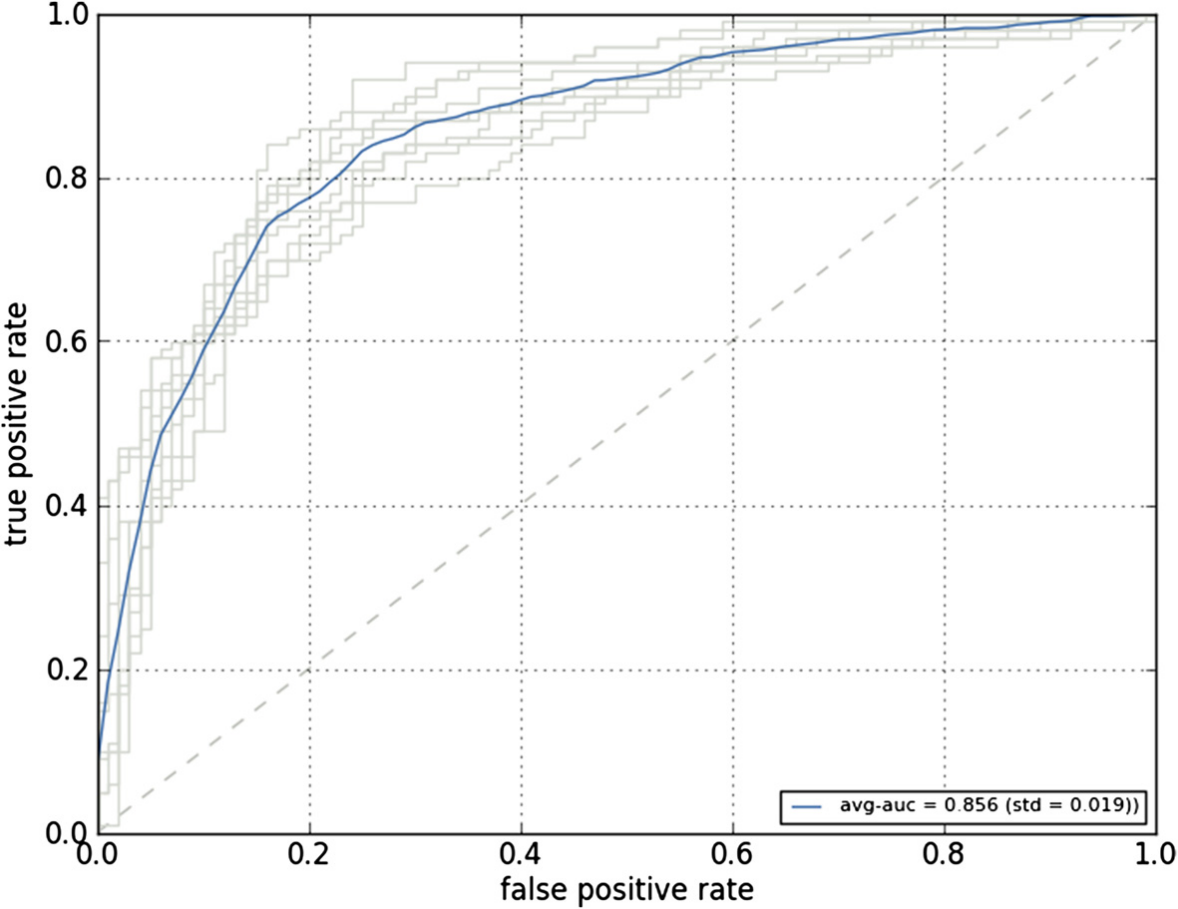
**Receiver operator characteristic (ROC) curve showing performance of a classifier trained for the yeast expression-level project.** The ROC curve shows the performance of a linear support vector machine classifier that was trained using the codon composition as features. Results for the 10 cross-validations are shown in gray, the average performance is shown in blue.

Using the *t*-statistics table in Figure 6, quick exploration of the amino acid composition reveals a preference for alanine, valine, and glycine in the high-expression class, whereas low-expression proteins contain relatively many asparagines and serines. The alanine and serine histograms in Figure 7, the features with minimal and maximal *t*-value respectively, indeed show shifted means of the class distributions. A classification performance, again using a linear support vector machine and 10-fold cross-validation, of 0.794 area under the ROC-curve (Additional file 1: Figure S8) showed good predictive capability of the amino acid composition. The predictive capability using the codon composition proved even better, resulting in a performance of 0.856 area under the ROC-curve (Figure 8).

For further exploration of the system, two additional example projects can be initiated. One entails protein subcellular localization in human, a data set of 2580 proteins categorized into 14 different subcellular locations as taken from [35]. The other is a solubility data set obtained from [36], consisting of 17.408 yeast proteins that are split into two equal sized classes: *soluble* and *insoluble*.

## Conclusion

SPiCE provides easy access to visualization and classification methods for a set of labeled protein sequences. After uploading a FASTA file with protein sequences and a label file with protein labels, the website can be used to calculate sequence-based features, to visualize the resulting feature matrix, and to train and test classifiers for predicting class labels, enabling quick exploration of sets of labeled proteins. The back-end software is made available for expert users to perform customized and computationally demanding tasks on a local computer.

## Availability and requirements

•**Project name:** SPiCE

•**URL:**http://helix.ewi.tudelft.nl/spice

•**Source code spice python package:**https://github.com/basvandenberg/spice

•**Source code spice web site:**https://github.com/basvandenberg/spiceweb

•**Web browsers:** Chrome, Firefox, Opera, Safari

•**Operating system:** Platform independent

•**Programming language:** Python 2.7

•**License:** GNU GPL v3

## Competing interests

The authors declare that they have no competing interests.

## Authors’ contributions

The software was developed by BAB under the supervision of DdR, MJTR, and JAR. BAB wrote the initial manuscript. All authors contributed to and approved the manuscript.

## Supplementary Material

Additional file 1 **Supplementary Information.** Showing the use of SPiCE by means of two example projects. Click here for file
