# Supplementary material for: SPiCE: a web-based tool for sequence-based protein classification and exploration
Source: BMC Bioinformatics. 2014 Mar 31;15:93. doi: 10.1186/1471-2105-15-93 (PMC4021553; doi:10.1186/1471-2105-15-93)
Supplement: Additional file 1 — Supplementary Information. Showing the use of SPiCE by means of two example projects. [file 1471-2105-15-93-S1.pdf]

SPiCE: Sequence-based Protein Classification and Exploration

Supplementary Data

Bastiaan A. van den Berg [1,3,4,*], Marcel J.T. Reinders [1,3,4], Johannes A.Roubos [2,3], and Dick de Ridder [1,3,4]

[1] Delft Bioinformatics Lab, Department of Intelligent Systems, Faculty Electrical Engineering, Mathematics and Computer Science, Delft University of Technology, Delft, The Netherlands,
[2] DSM Biotechnology Center, Delft, The Netherlands,
[3] Netherlands Bioinformatics Centre, Nijmegen, The Netherlands,
[4] Kluyver Centre for Genomics of Industrial Fermentation, Delft, The Netherlands

* to whom correspondence should be addressed

Aspergillus niger high-level secretion example

This example project uses the data set from our previous work[1], composed of 345 *Aspergillus niger* proteins. All proteins in the data set have a predicted signal peptide (SignalP 3.0) and do not contain an ER retention signal or predicted transmembrane regions (TMHMM, phobius). The proteins were over-expressed behind a strong constitutive promoter, to test for successful high-level production and secretion. A protein was labeled successful (*pos*, 178 proteins) in case of a visible band on gel, and unsuccessful (*neg*, 167 proteins) otherwise.

The SPiCE website was used to calculate the amino acid composition, which was in turn used to: obtain *t*-statistics (Fig. S1), visualize histograms for the 4 features with the highest absolute *t*-value (Fig. S2), visualize the clustered heatmap of the full feature matrix (Fig. S3), and obtain classification performance of a linear SVM (Fig. S4).

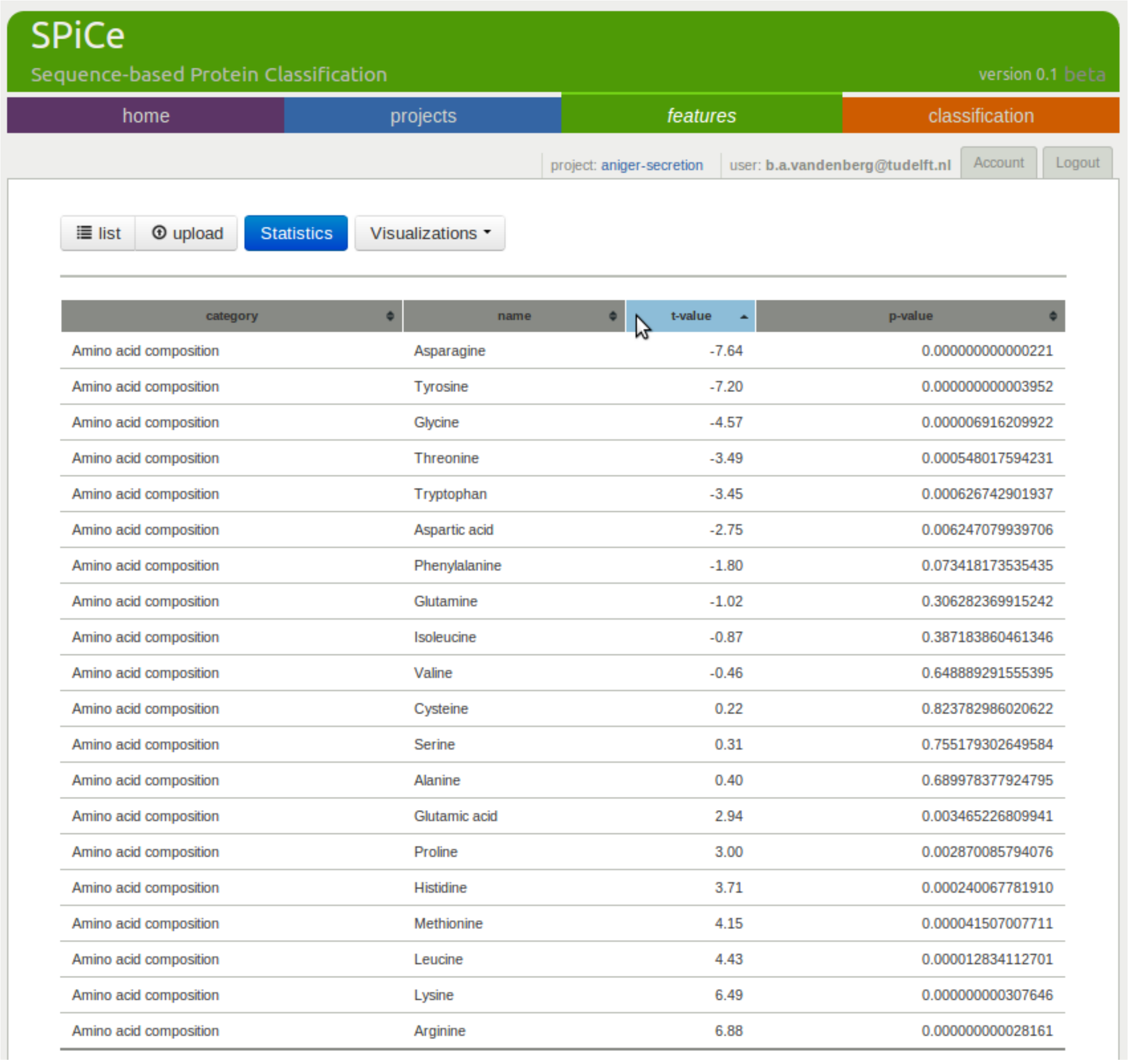

category	name	t-value	p-value
Amino acid composition	Asparagine	-7.64	0.000000000000221
Amino acid composition	Tyrosine	-7.20	0.000000000003952
Amino acid composition	Glycine	-4.57	0.000006916209922
Amino acid composition	Threonine	-3.49	0.000548017594231
Amino acid composition	Tryptophan	-3.45	0.000626742901937
Amino acid composition	Aspartic acid	-2.75	0.006247079939706
Amino acid composition	Phenylalanine	-1.80	0.073418173535435
Amino acid composition	Glutamine	-1.02	0.306282369915242
Amino acid composition	Isoleucine	-0.87	0.387183860461346
Amino acid composition	Valine	-0.46	0.648889291555395
Amino acid composition	Cysteine	0.22	0.823782986020622
Amino acid composition	Serine	0.31	0.755179302649584
Amino acid composition	Alanine	0.40	0.689978377924795
Amino acid composition	Glutamic acid	2.94	0.003465226809941
Amino acid composition	Proline	3.00	0.002870085794076
Amino acid composition	Histidine	3.71	0.000240067781910
Amino acid composition	Methionine	4.15	0.000041507007711
Amino acid composition	Leucine	4.43	0.000012834112701
Amino acid composition	Lysine	6.49	0.000000000307646
Amino acid composition	Arginine	6.88	0.000000000028161

Fig S1: Screenshot of the *A. niger* high-level secretion example project, showing the *t*-test table for the amino acid composition features sorted by their *t*-value.

[1] van den Berg, Bastiaan A., et al. "Exploring Sequence Characteristics Related to High-Level Production of Secreted Proteins in Aspergillus niger." *PloS ONE* 7.10 (2012): e45869.

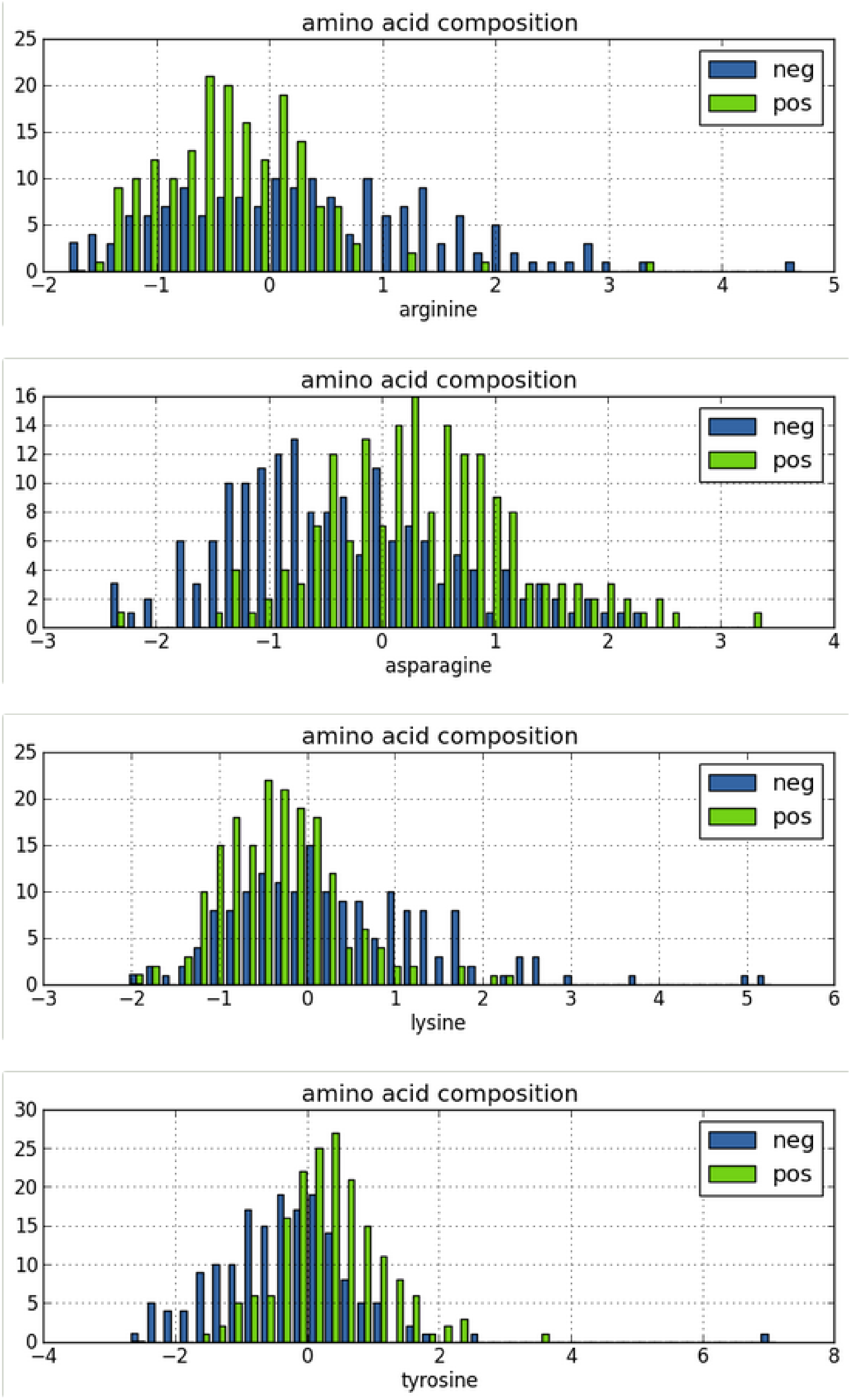

Fig S2: Screenshot of the *A. niger* high-level secretion example project, showing the histograms of the 4 amino acid composition features with the highest absolute *t*-values.

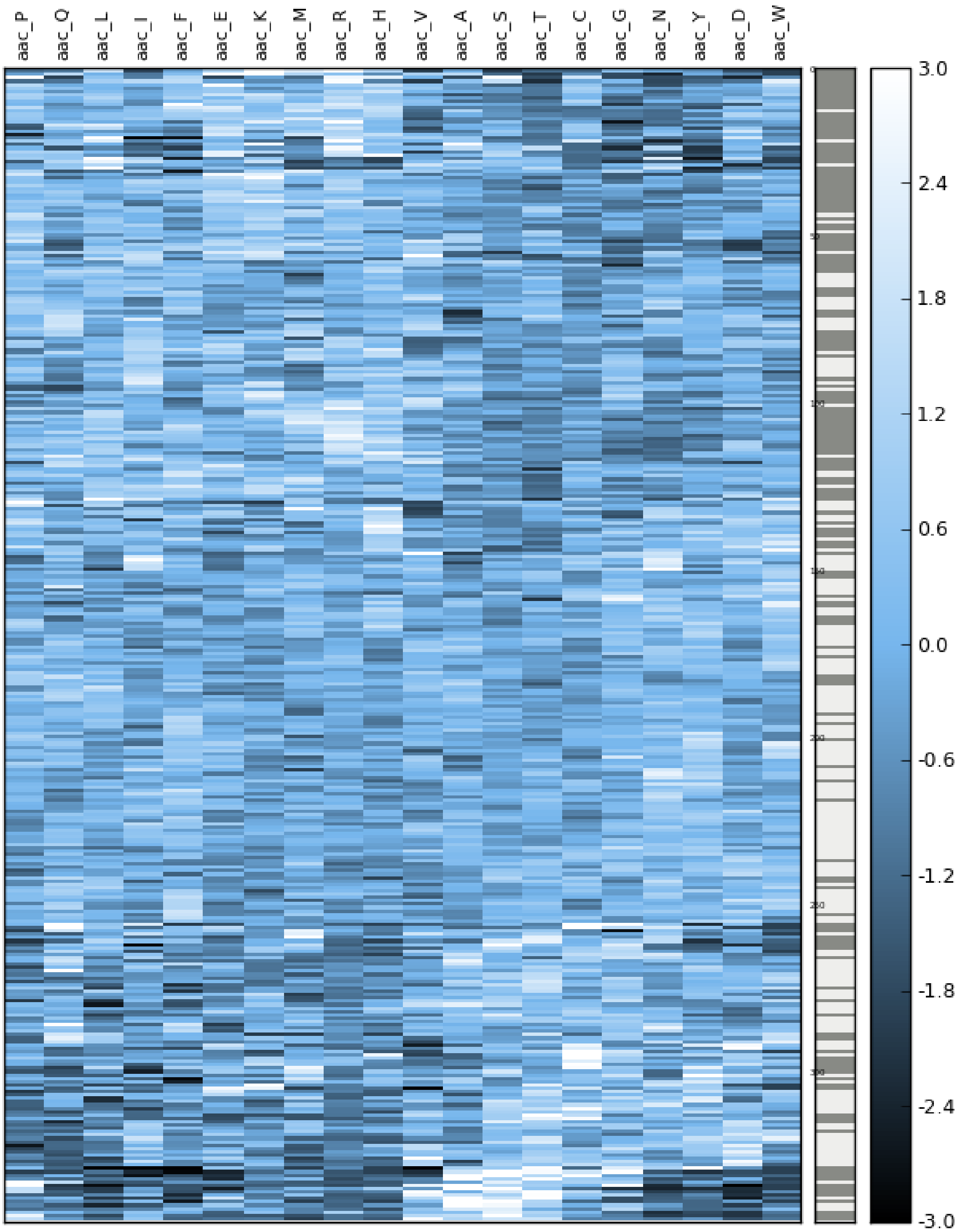

Fig S3: Heat map showing the feature matrix with the 245 proteins of the *A. niger* high-level secretion example project as rows and their amino acid composition feature values as columns. Both the rows and the columns are hierarchically clustered. Additionally, the protein labels are shown in the right column, with gray for unsuccessful high-level secretion and white for successful high-level secretion. The fact that the labels form clusters indicates that these features can be useful for classification.

Classifier performance scores

CV	accuracy	average_precision	f1	precision	recall	roc_auc
0	0.657	0.782	0.684	0.650	0.722	0.768
1	0.771	0.730	0.778	0.778	0.778	0.791
2	0.714	0.901	0.737	0.700	0.778	0.882
3	0.800	0.857	0.821	0.762	0.889	0.899
4	0.800	0.908	0.788	0.867	0.722	0.879
5	0.794	0.831	0.811	0.750	0.882	0.844
6	0.735	0.788	0.780	0.667	0.941	0.785
7	0.853	0.794	0.857	0.882	0.833	0.885
8	0.794	0.701	0.811	0.789	0.833	0.802
9	0.735	0.780	0.791	0.680	0.944	0.833
avg/std	0.77 / 0.05	0.81 / 0.06	0.79 / 0.05	0.75 / 0.08	0.83 / 0.08	0.84 / 0.05

ROC curve

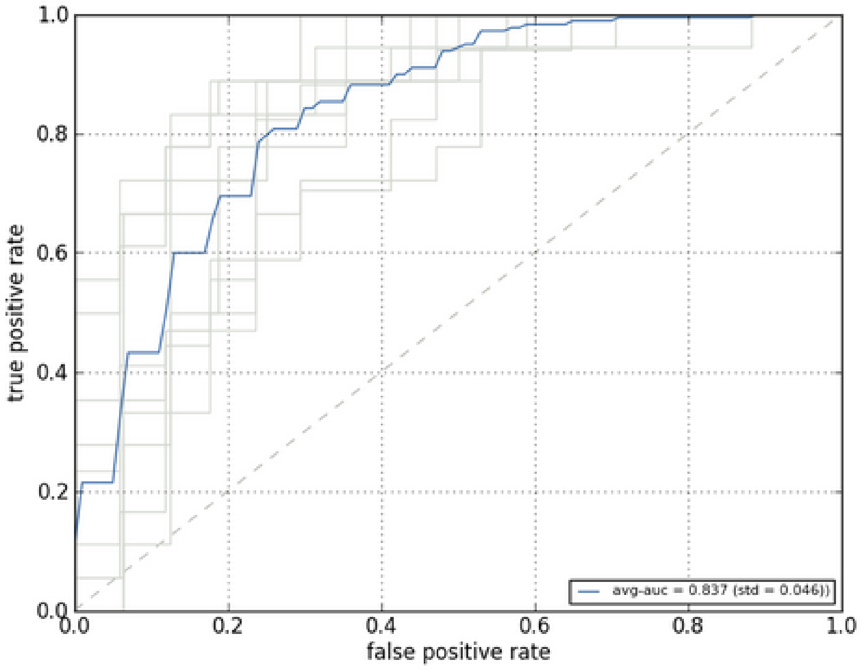

Fig S4: This screenshot shows classification results for the *A. niger* high-level secretion example project. In this case, a 10-fold cross-validation classification performance of 0.837 area under the ROC-curve is achieved with a linear SVM using the amino acid composition as features.

Yeast expression example

The yeast expression example is constructed from a yeast protein expression data set[2]. CDHit[3] was used to reduce sequence redundancy so that none of the protein sequences share more than 35% sequence identity. The list of proteins was sorted by their observed protein molecules per cell and the top and bottom thousand proteins were taken as the *high* and *low* class.

The SPiCE website was used to calculate the amino acid composition, which were in turn used to: obtain *t*-statistics (Fig. S5), visualize histograms for the 5 features with the highest absolute *t*-value (Fig. S6), visualize the clustered heatmap of the full feature matrix (Fig. S7), and to obtain classification performance of a linear SVM (Fig. S8).

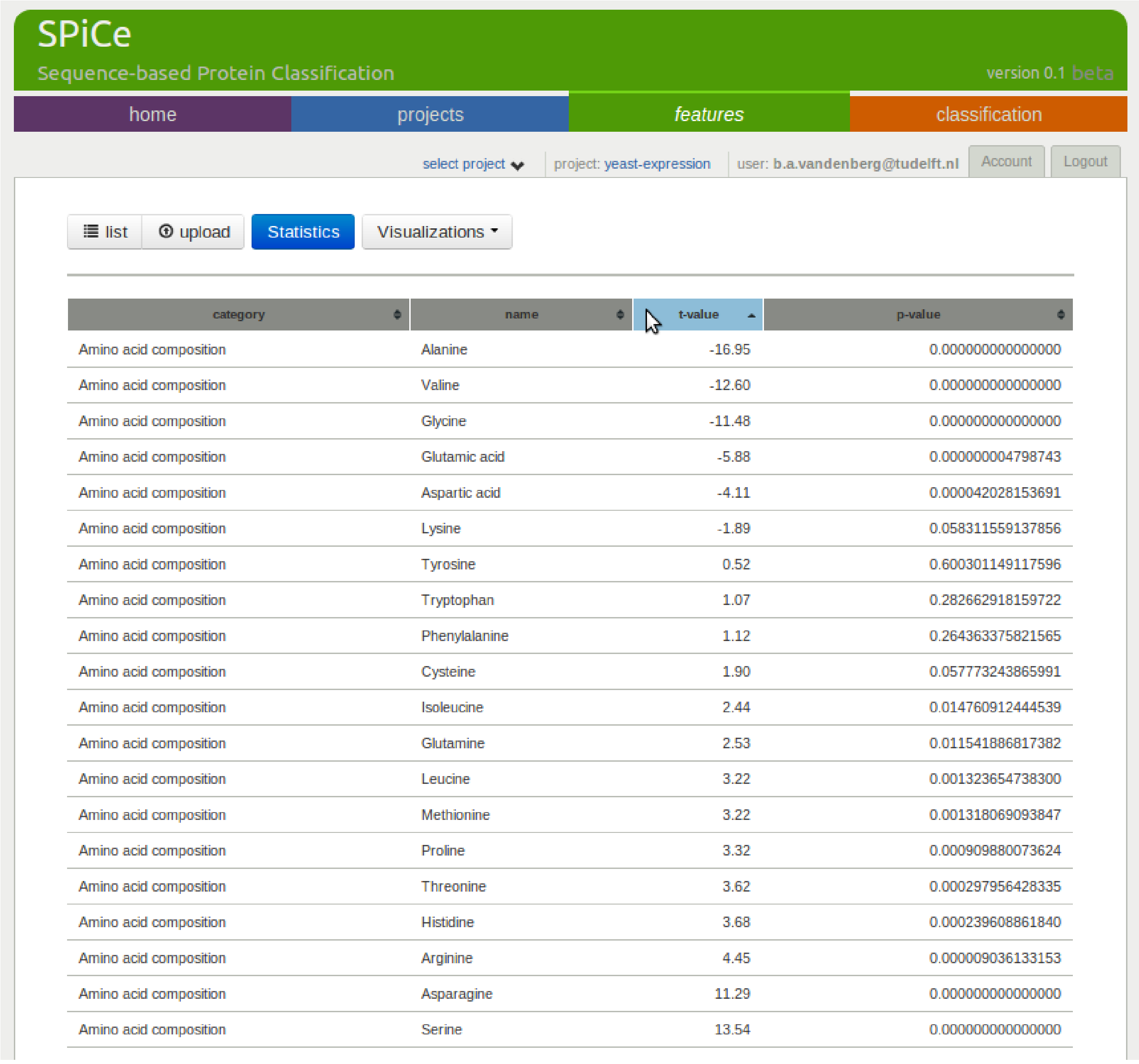

category	name	t-value	p-value
Amino acid composition	Alanine	-16.95	0.000000000000000
Amino acid composition	Valine	-12.60	0.000000000000000
Amino acid composition	Glycine	-11.48	0.000000000000000
Amino acid composition	Glutamic acid	-5.88	0.000000004798743
Amino acid composition	Aspartic acid	-4.11	0.000042028153691
Amino acid composition	Lysine	-1.89	0.058311559137856
Amino acid composition	Tyrosine	0.52	0.600301149117596
Amino acid composition	Tryptophan	1.07	0.282662918159722
Amino acid composition	Phenylalanine	1.12	0.264363375821565
Amino acid composition	Cysteine	1.90	0.057773243865991
Amino acid composition	Isoleucine	2.44	0.014760912444539
Amino acid composition	Glutamine	2.53	0.011541886817382
Amino acid composition	Leucine	3.22	0.001323654738300
Amino acid composition	Methionine	3.22	0.001318069093847
Amino acid composition	Proline	3.32	0.000909880073624
Amino acid composition	Threonine	3.62	0.000297956428335
Amino acid composition	Histidine	3.68	0.000239608861840
Amino acid composition	Arginine	4.45	0.000009036133153
Amino acid composition	Asparagine	11.29	0.000000000000000
Amino acid composition	Serine	13.54	0.000000000000000

Fig S5: Screenshot of the yeast-expression example project, showing the table with t-values for the amino acid composition features.

[2] Ghaemmaghami, Sina, et al. "Global analysis of protein expression in yeast."*Nature* 425.6959 (2003): 737-741.
[3] http://weizhong-lab.ucsd.edu/cdhit_suite/cgi-bin/index.cgi?cmd=cd-hit

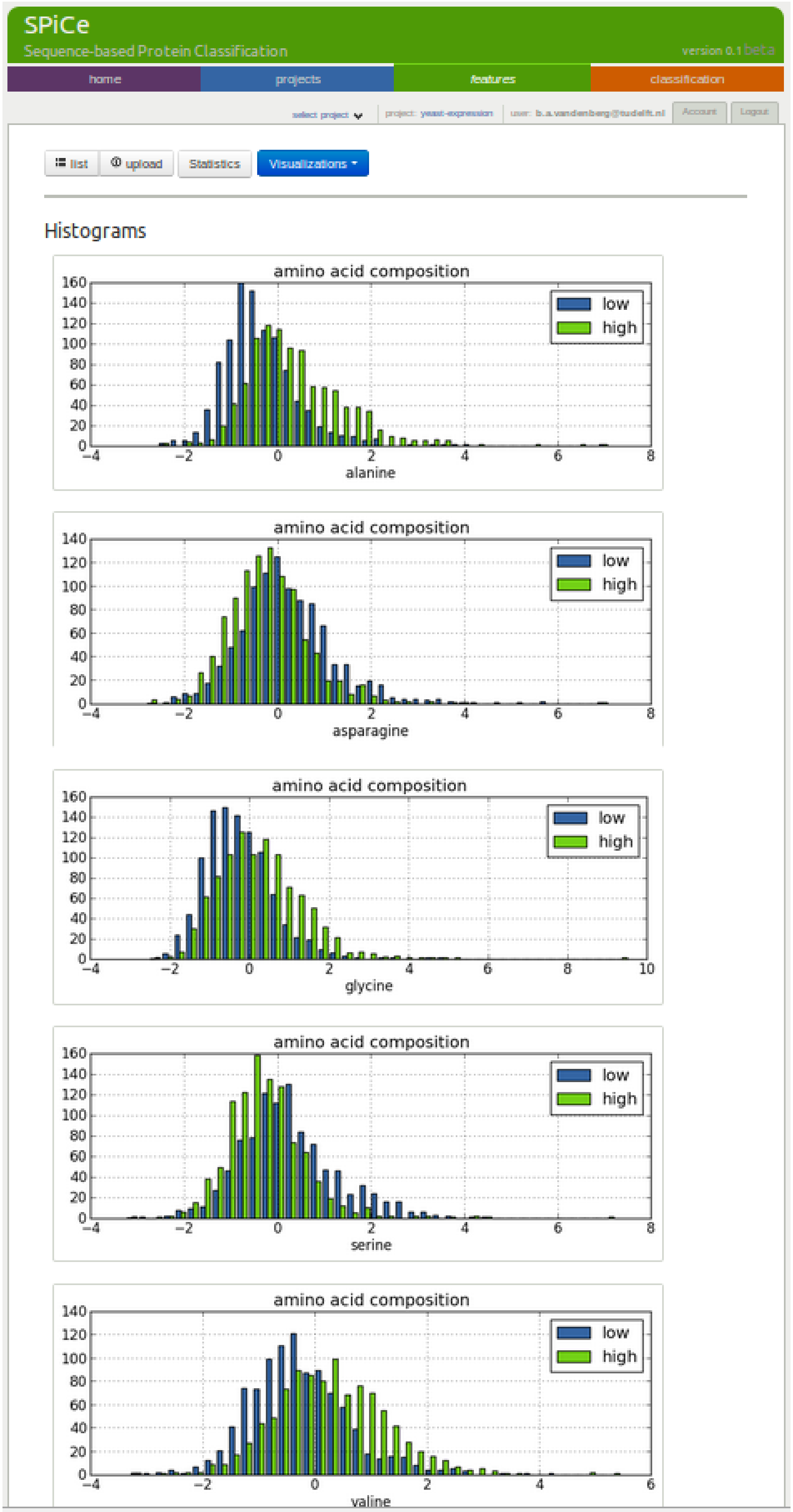

Fig S6: Screenshot of the yeast-expression example project, showing the histograms of the 5 amino acid composition features with the highest absolute *t*-values.

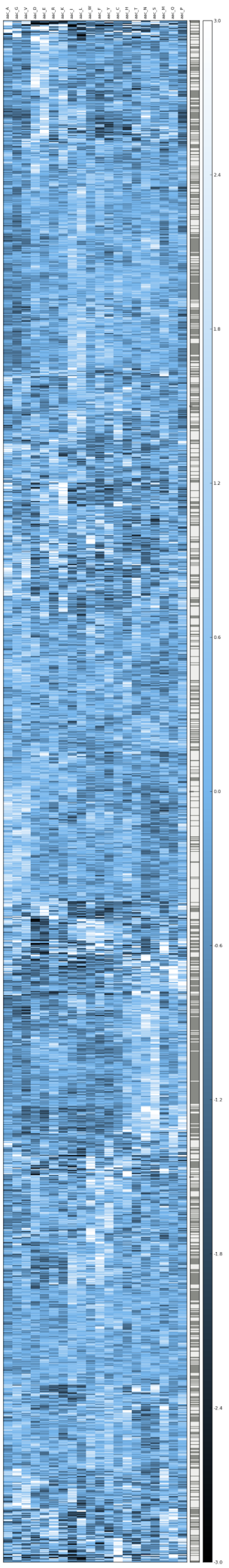

Fig S7: Heat map showing the hierarchically clustered feature matrix with the 2000 proteins of the yeast-expression example project as rows and their amino acid composition feature values as columns. The protein labels are in the right column, with gray for low expression and white for high expression.

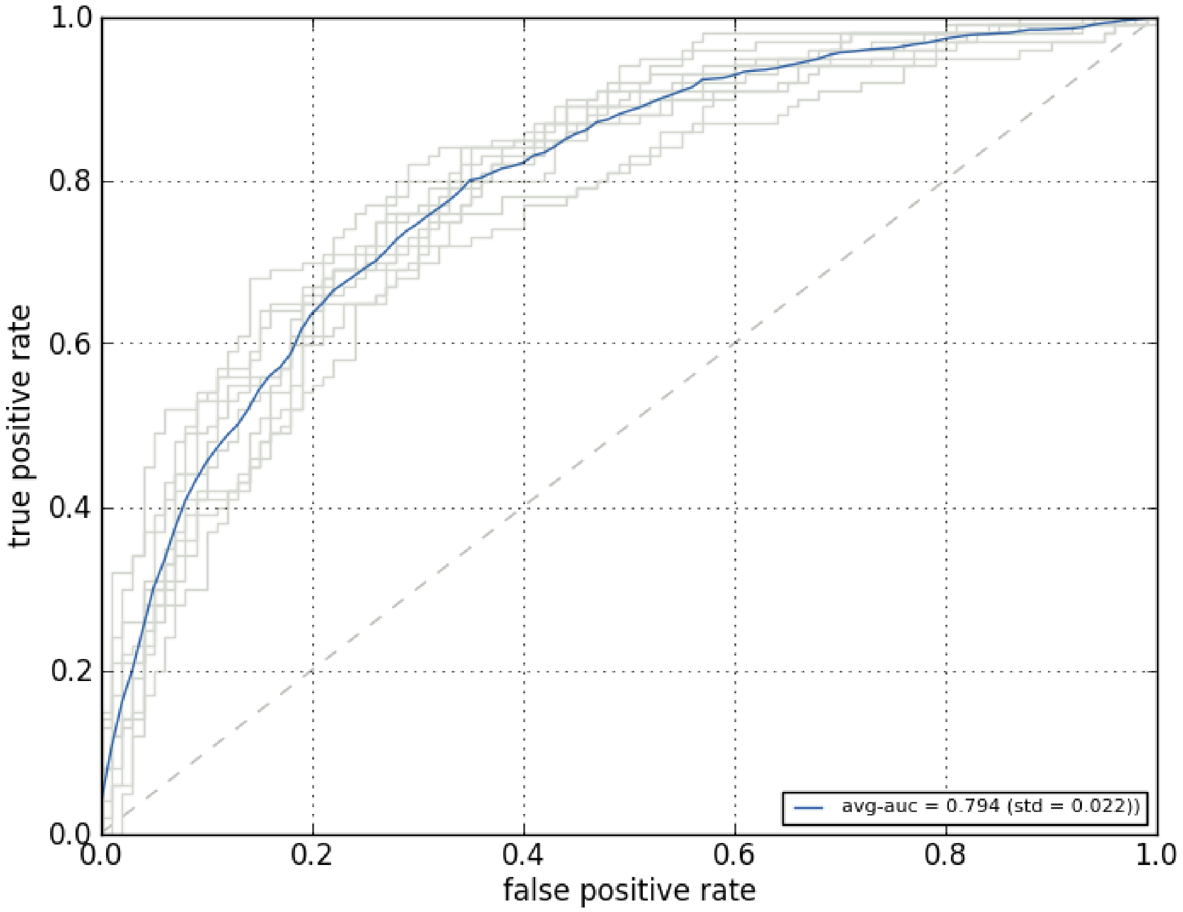

Fig S8: A classification performance of 0.794 area under the ROC-curve is achieved with a linear SVM using the amino acid composition as features.
